# LAPTM4B Allele *2 Is a Marker of Poor Prognosis for Gallbladder Carcinoma

**DOI:** 10.1371/journal.pone.0045290

**Published:** 2012-09-12

**Authors:** Guojun Zhai, Kaowen Yan, Xiaoxu Ji, Wenrui Xu, Jiuling Yang, Fuxia Xiong, Jing Su, Michael A. McNutt, Hua Yang

**Affiliations:** 1 Department of Interventional Radiology and Vascular Surgery, Peking University Third Hospital, Beijing, China; 2 Department of Cell Biology, School of Basic Medical Sciences, Peking University, Beijing, China; 3 Department of General Surgery, Peking University Third Hospital, Beijing, China; 4 Department of Pharmacology, Loma Linda University, Loma Linda, California, United States of America; 5 Department of Pathology, School of Basic Medical Sciences, Peking University, Beijing, China; Pontificia Universidad Catolica de Chile, Chile

## Abstract

**Background:**

Lysosomal protein transmembrane 4 beta (LAPTM4B) is a novel cancer-related gene which has two alleles designated LAPTM4B*1 and LAPTM4B*2. In this study we investigated the correlation of LAPTM4B genotype with prognosis and clinicopathologic features in patients who had undergone curative resection for gallbladder carcinoma (GBC).

**Methodology/Principal Findings:**

PCR assay was performed to determine the LAPTM4B genotype in 85 patients. The correlation of LAPTM4B genotype with clinicopathologic parameters was assessed with the Chi-squared test. Differences in patient survival were determined by the Kaplan–Meier method. Multivariate analysis of prognostic factors was carried out with Cox regression analysis. Patients with LAPTM4B *2 had both significantly shorter overall survival (OS) and shorter disease-free survival (DFS) (both P<0.001). Multivariate analysis showed that LAPTM4B genotype is a prognostic factor for OS and DFS (both P<0.001).

**Conclusions/Significance:**

LAPTM4B allele *2 is a risk factor associated with poor prognosis in patients with resected GBC, and LAPTM4B status may be therefore be useful preoperatively as an adjunct in evaluation of the operability of GBC.

## Introduction

Gallbladder carcinoma (GBC) is a highly aggressive neoplasm with a poor prognosis [1], and it is the fifth most common tumor of the digestive tract [2]. In 2008 the incidence of GBC was 2.4 per 100,000 individuals and the mortality rate was 1.6 per 100,000 in developed areas [3]. Due to the gallbladder's anatomic position and non-specific symptoms associated with GBC, a high proportion of gallbladder carcinomas are advanced at the time of diagnosis, leading to less than 10% overall 5-year survival and less than 5% survival for tumors of TNM stage III–IV [4]–[6]. In spite of recent improvements in surgery and therapeutic modalities such as chemotherapy and radiotherapy, it is difficult to successfully treat gallbladder carcinoma. Therefore, it is necessary to find effective early diagnostic and therapeutic markers.

Lysosomal protein transmembrane-4 beta (*LAPTM4B*) is a cancer-related gene which was originally cloned from hepatocellular carcinoma tissues in our laboratory [7]. It has been reported that LAPTM4B protein is significantly up-regulated in a wide variety of cancers including hepatocellular carcinoma, extra-hepatic cholangiocarcinoma, breast cancer, endometrial carcinoma, cervival carcinoma and ovarian cancer as well as gallbladder carcinoma. This overexpression is significantly correlated with prognosis [8]–[16]. The LAPTM4B gene is amplified in breast cancer and its amplification is related to breast carcinoma recurrence [17]. Moreover, transfection of LAPTM4B cDNA promotes proliferation, migration, invasion and multidrug resistance in HCC and GBC cells [18]–[22], and knockdown of LAPTM4B by RNAi reverses multiple malignant phenotypes [19], [22]. These data show the LAPTM4B gene plays a role in many types of neoplasia, and led us to consider whether it may serve as a prognostic factor in GBC.

The LAPTM4B gene has two alleles (GenBank No. AY219176 and AY219177). LAPTM4B allele *1 (LAPTM4B*1) contains a particular 19-bp sequence, and in allele *2 (LAPTM4B*2) this sequence is duplicated and tandemly arranged in the first exon of the LAPTM4B gene [23]. Previous studies have reported that allelic variant LAPTM4B*2 is associated with genetic susceptibility to gallbladder carcinoma, hepatocellular carcinoma, gastric cancer and colon cancer [23]–[27], and these data suggest that LAPTM4B gene polymorphism plays an important role in carcinogenesis. As reported in our previous study, we found that the allelic frequencies of the LAPTM4B*2 allele were 37.9% in the GBC group and 24.8% in the control group, representing a significant difference between these two groups (P<0.001), and suggesting that the LAPTM4B*2 allele is associated with significantly increased risk of gallbladder carcinoma [27]. In addition, we recently showed that LAPTM4B allele *2 is a marker of poor prognosis in hepatocellular carcinoma [28]. However, it is unclear whether there is a correlation of LAPTM4B gene polymorphism with prognosis in GBC. In the current study we therefore evaluated the genotype in patients who had GBC surgery for attempted curative resection.

## Materials and Methods

### Patients

Blood samples were obtained from 85 GBC patients who were hospitalized and underwent surgical resection in Third Hospital Affiliated with Peking University from January 2000 to December 2009. All patients who had attempted curative resection for GBC in Third Hospital during this period were included in this study and there were no other selection criteria for inclusion in this study. This was a retrospective study with overall survival and disease free survival as its two endpoints. Among the patients 23 were male and 62 were female, which is not unexpected as F:M ratios in areas of high GBC incidence may be as high as 6∶1[29], and the mean patient age was 44.6 (range 25–78). TNM staging was carried out for each patient according to AJCC-UICC guidelines (AJCC_UICC, 5th edition, 1997). Clinicopathologic features in this group of patients including tumor size, lymph node metastasis, histopathologic differentiation and TNM stage are summarized in Table 1. The Institutional Ethics Committee of Peking University approved this study before investigation was begun, and all patients gave written informed consent for participation.

**Table 1:** Relationship between LAPTM4B genotype and clinicopathologic features in GBC patients.

Variables	Patients	LAPTM4B genotype			*P^a^*
		^*^1/1	^*^1/2	^*^2/2	
Gender					0.987
Male	23	8	12	3	
Female	62	22	31	9	
Age (years)					0.937
<60	49	18	24	7	
≥60	36	12	19	5	
Tumor diameter					0.521
<2 cm	44	18	20	6	
≥2 cm	41	12	23	6	
Histopathologic differentiation					0.008
Well differentiated	28	16	9	3	
Moderately differentiated	29	10	17	2	
Poorly differentiated	28	4	17	7	
Lymph node metastasis					<0.001
No	61	29	28	4	
Yes	24	1	15	8	
TNM stage					<0.001
I–II	28	23	4	1	
III–IV	57	7	39	11	

LAPTM4B, Lysosomal protein transmembrane 4 beta; GBC, Gallbladder carcinoma; *a*, Chi-square test.

### DNA extraction

Human peripheral blood samples were collected in EDTA tubes and kept at −20°C. Blood was thawed in a water bath at room temperature and genomic DNA was isolated from 200 ul of each blood sample using the NucleoSpin Blood genomic DNA extraction kit (Macherey-Nagel, Duren, Germany) for PCR assay.

### PCR analysis

LAPTM4B genotype was determined by PCR analysis using the specific primers F (forward): 5′ GCCGACTAGGGGACTGGCGGA 3′ (nt 72–92) and R (reverse): 5′ CGAGAGCTCCGAGCTTCTGCC 3′ (nt 255–275). PCR assay was carried out in a 20 ul reaction mixture containing 0.5 U Taq DNA polymerase (NEB, Beijing, China) using 1 ul of template DNA of 100 ng/ul. The PCR conditions were 95°C denaturation for 5 min, 35 cycles of 30 s at 94°C, 30 s at 60°C and 30 s at 72°C, followed by extension at 72°C for 10 min. PCR products were analyzed by electrophoresis in a 2% agarose gel and visualized with ethidium bromide.

### Statistical analysis

The Chi-square test was used to demonstrate differences in the genotypic distribution of LAPTM4B and categorical variables. Differences in patient survival were determined by the Kaplan–Meier method and the log-rank test. Variables which showed significant correlation by the Kaplan–Meier were used in Cox regression analysis (Proportional hazard model) for multivariate analysis of prognostic factors. The statistical software package SPSS10.0 (SPSS Inc., Chicago, IL) was employed for all analysis. P values of <0.05 were defined as statistically significant.

## Results

### Genotypes of the LAPTM4B gene

**Figure 1:**
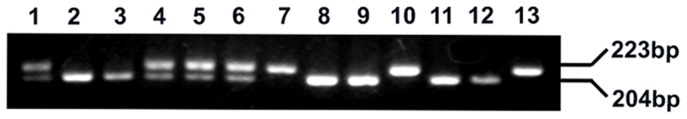
LAPTM4B genotyping. Analysis by separation with 2% agarose gel electrophoresis. Lanes 1, 4, 5, 6: genotype*1/2; lanes 2, 3, 8, 9, 11, 12: genotype*1/1; lanes 7, 10, 13: genotype *2/2.

Three different genotypic LAPTM4B polymorphisms designated LAPTM4B*1, LAPTM4B*2 and LAPTM4B*1/*2 were identified by PCR assay. As shown in Figure 1, genotype *1/1 is represented by a 204-bp band, *2/*2 is represented by a 223-bp band, and genotype *1/*2 shows both of these bands. All bands were identified with PCR using specific primers and separated by electrophoresis in a 2% agarose gel.

### Genotypes of LAPTM4B and clinical parameters

These 85 patients who underwent surgery at Third Hospital Afiliated with Peking University from 2000 to 2009 were followed clinically, with follow-up ranging from 2 to 95 months (median 35 months). As of December 31, 2010 which was the end date for follow up, 19 (22.4%) patients were alive, and 66 (77.6%) patients had died of disease. We found that genotype *2 of the LAPTM4B gene was significantly associated with poor histopathologic differentiation, higher TNM stage and lymph node metastasis (Table 1; P<0.05), but not with age, gender, or tumor size (Table 1; P>0.05).

### LAPTM4B genotype and GBC prognosis

**Figure 2:**
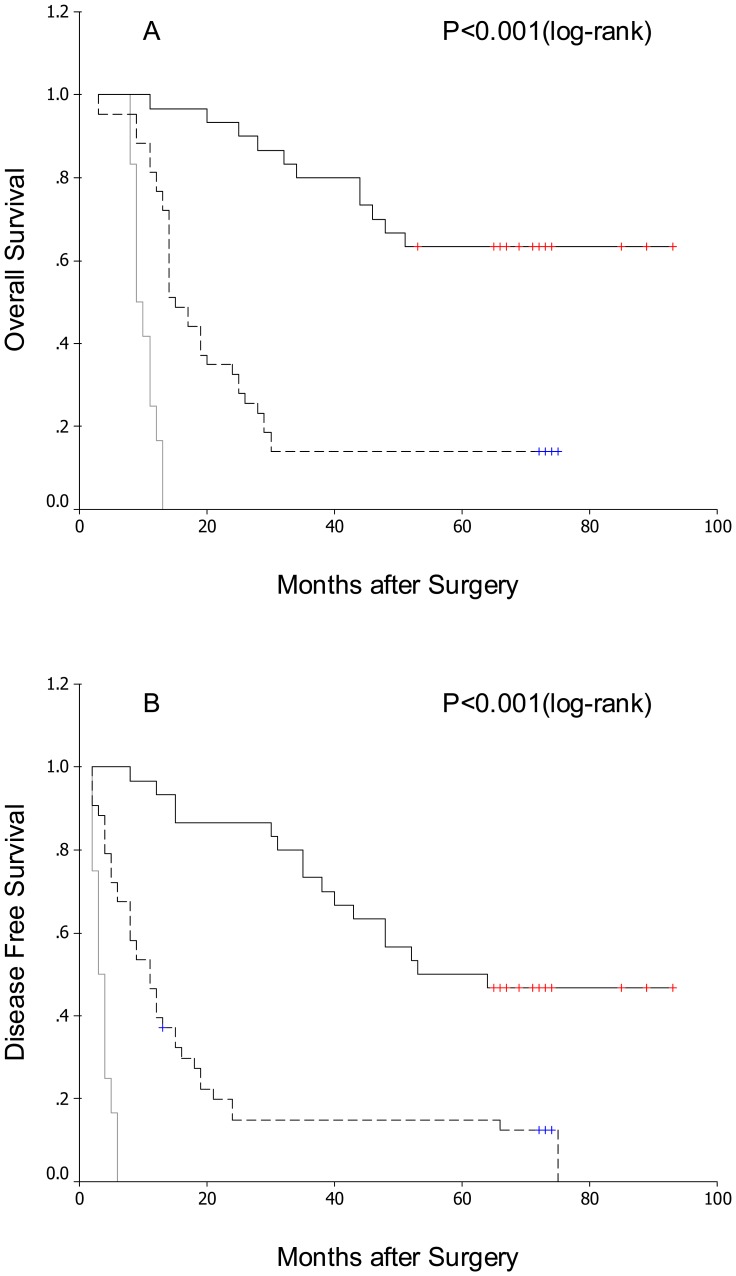
Comparison of survival in patients after surgical resection of GCB based on evaluation of LAPTM4B genotypes. LAPTM4B genotype *1/1, black solid line; LAPTM4B genotype *1/2, black dashed line; LAPTM4B genotype *2/2, grey solid line. (A) Overall survival after surgery. (B) Disease-free survival after surgery.

Using the Kaplan–Meier method and the log-rank test, genotype *2 of the LAPTM4B gene showed correlation both with shorter disease-free survival and shorter overall survival in these 85 patients (Fig. 2A and B, and Table 2; both P<0.001). In addition, as expected survival benefit was also found in patients with lower TNM stage, lower grade histopathologic differentiation and absence of lymph node metastasis both for overall and disease-free survival (Table 2; P<0.05). No other clinicopathologic features showed predictive value in this analysis (Table 2; P>0.05).

**Table 2:** Univariate Survival Analysis of OS and DFS in 85 patients with GBC.

Variables	No.of cases	OS			DFS		
		Mean±SE (month)	95% CI	*P ^a^*	Mean±SE (month)	95% CI	*P ^a^*
Gender				0.273			0.388
Male	23	40±6	(28–51)		34±6	(23–46)	
Female	62	38±4	(30–46)		31±4	(22–40)	
Age (years)				0.918			0.758
<60	49	41±5	(31–50)		34±5	(24–45)	
≥60	36	40±6	(29–52)		31±5	(20–41)	
Tumor diameter				0.929			0.737
<2 cm	44	40±5	(30–50)		31±5	(22–41)	
≥2 cm	41	39±5	(29–50)		34±6	(23–44)	
Lymph node metastasis				<0.001			<0.001
No	61	52±5	(43–60)		43±5	(34–52)	
Yes	24	13±1	(10–15)		7±1	(5–10)	
Histopathologic differentiation				0.004			0.008
WD	28	56±7	(42–69)		45±6	(32–57)	
MD	29	39±6	(27–50)		34±6	(21–46)	
PD	28	24±5	(15–33)		18±5	(8–27)	
TNM stage				<0.001			<0.001
I–II	28	75±6	(64–86)		65±6	(54–76)	
III–IV	57	23±3	(17–29)		17±3	(11–23)	
LAPTM4B genotype				<0.001			<0.001
^*^1/1	30	72±5	(61–82)		62±6	(51–73)	
^*^1/2	43	25±3	(18–31)		20±4	(12–27)	
^*^2/2	12	10±1	(9–11)		4±0	(3–4)	

LAPTM4B, Lysosomal protein transmembrane 4 beta; GBC, Gallbladder carcinoma; OS, overall survival; DFS, disease-free survival; *a*, Log-rank test.

### LAPTM4B Genotype is a prognostic maker in patients who have undergone curative resection for GBC

Multivariate Cox regression analysis revealed that LAPTM4B genotype (RR, 2.809, 95%CI, 1.506-5.240, P = 0.001), lymph node metastasis (RR, 2.937, 95%CI, 1.519-5.679, P = 0.001) and TNM stage (RR, 2.857, 95%CI, 1.214-6.721, P = 0.016) are prognostic markers for overall survival in patients with GBC (Table 3; P<0.05). LAPTM4B genotype (RR, 2.740, 95%CI, 1.549-4.846, P = 0.001) and lymph node metastasis (RR, 2.604, 95%CI, 1.368-4.955, P = 0.004) are also prognostic markers for disease-free survival in these patients (Table 3; P<0.05).

**Table 3:** Multivariate Survival Analysis of OS and DFS in 85 patients with GBC.

Variables	OS			DFS		
	RR	95% CI	*P^a^*	RR	95% CI	*P^a^*
TNM stage	2.857	1.214–6.721	0.016	1.926	0.946–3.919	0.071
Lymph node metastasis	2.937	1.519–5.679	0.001	2.604	1.368–4.955	0.004
Histopathologic differentiation	1.262	0.889–1.792	0.193	1.274	0.915–1.774	0.151
LAPTM4B genotype	2.809	1.506–5.240	0.001	2.740	1.549–4.846	0.001

LAPTM4B, Lysosomal protein transmembrane 4 beta; GBC, Gallbladder carcinoma; OS, overall survival; DFS, disease-free survival; RR, relative risk; CI, confidence interval; *a*, Cox regression test.

## Discussion

In this study, we tested for LAPTM4B genotype by PCR assay in 85 patients who had surgical resection for gallbladder carcinoma. We found that LAPTM4B *2 is significantly correlated with poor histopathologic differention, higher TNM stage and lymph node metastasis, but this allele showed no relationship to patient gender, patient age or tumor size. Moreover, patients with LAPTM4B *2 (alleles *2/2 or *1/2) had significantly poorer overall survival (OS) and disease-free survival (DFS) as compared to patients with LAPTM4B *1/1.

This is the first time correlation of LAPTM4B genotype with prognosis and clinicopathologic features has been demonstrated in gallbladder carcinoma. This association of genotype with clinicopathologic findings and prognosis is consistent with the putative role LAPTM4B plays in carcinogenesis and tumor progression. Previous studies which have shown upregulation and/or amplification of the LAPTM4B gene in a variety of human cancers, and this together with experimental evidence showing that malignant phenotypic features can be induced or reversed by transfection or knockdown respectively of LAPTM4B strongly suggests that this gene plays a fundamental role in the neoplastic mechanism(s) of these tumors [17], [19], [22]. There are some recent findings which may in part serve to illustrate how LAPTM4B functions in tumors which harbor this gene. For example, overexpression of LAPTM4B may activate the AKT signaling pathway and proto-oncogenes including c-fos, c-myc, and c-jun and thus promote malignant transformation [22]. In addition, knock-down of LAPTM4B or mutation of the PPRP motif in the N-terminal region of LAPTM4B attenuates its roles in tumorigenesis and metastasis [19], [22].

The allele LAPTM4B*1 (GenBank No. AY219176) differs from allele LAPTM4B*2 (GenBank No. AY219177) in that it contains a particular single 19-bp sequence, whereas LAPTM4B *2 contains two of these sequences in a tight tandem array in the 5′ UTR of exon 1. LAPTM4B*1 produces a 35kD protein while LAPTM4B*2 produces a 40kD protein with an extra 53 amino acids at the N terminus which are not present in the protein produced by LAPTM4B*1. This difference in proteins is due to the 19-bp difference in the first exon of the gene which alters the ORF. Previous studies have shown that LAPTM4B polymorphisms are associated with increased risk for gallbladder carcinoma, gastric cancer, colon cancer and liver cancer in Chinese patients [23]–[27]. The allelic frequencies of the *2 allele were 33.9%, 33.2%, and 40.1% in individuals with gastric, colon, and lung cancer, respectively, which were significantly higher than those of corresponding healthy controls.[24], [25] In view of the latter and given the extensive evidence of LAPTM4B's involvement in neoplasia, together with this gene's demonstrated relation to histologic grade and tumor recurrence in some cancers, it is likely that this 19-bp sequence plays an important role in genetic transcriptional regulation or that the extra 53 amino acids at the N terminus produced by LAPTM4B* 2 may influence physiological activity and function in tumor cells.

Although the exact molecular mechanisms which underlie the function of LAPTM4B in gallbladder carcinogenesis have not as yet been worked out, LAPTM4B*2 may be an oncogene or play an important role in cell cycle control. However, the clear association of LAPTM4B *2 with poor histopathologic differentiation, higher TNM stage and presence of lymph node metastasis argues that it is a significant factor in the highly malignant phenotype of gallbladder carcinoma and therefore warrants further investigation. In addition, it is a prognostic factor for postoperative survival in patients who have undergone curative surgical resection for GBC. Therefore, it is possible that LAPTM4B *2 will be useful as an adjunct for evaluation of GBC prior to embarking upon curative surgical resection.
